# Supplementary material for: Association of Mutation Profiles with Postoperative Survival in Patients with Non–Small Cell Lung Cancer
Source: Cancers (Basel). 2020 Nov 21;12(11):3472. doi: 10.3390/cancers12113472 (PMC7700403; doi:10.3390/cancers12113472)
Supplement: Supplementary file 1 [file cancers-12-03472-s001.pdf]

cancers

MDPI

Article

Association of Mutation Profiles with Postoperative Survival in Patients with Non–Small Cell Lung Cancer

Taichiro Goto, Kei Kunimasa, Yosuke Hirotsu, Takahiro Nakagomi, Yujiro Yokoyama, Rumi Higuchi, Sotaro Otake, Toshio Oyama, Kenji Amemiya, Hitoshi Mochizuki, and Masao Omata

Supplementary Materials

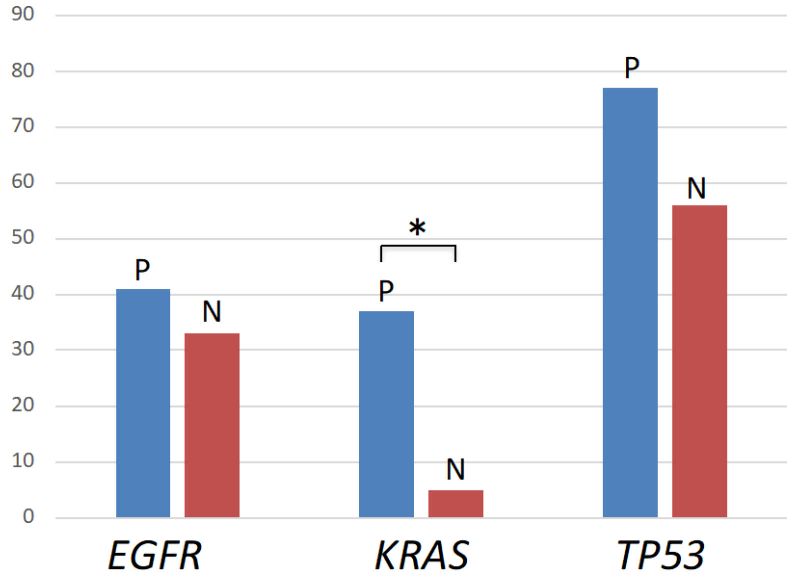

Figure S1. Pathogenic versus nonpathogenic mutations. The distribution of pathogenic and nonpathogenic mutations in epidermal growth factor receptor (*EGFR*), *KRAS*, and tumor protein p53 (*TP53*) is shown. *KRAS* mutations were significantly more frequently pathogenic than *EGFR* and *TP53*. Blue and red bars denote pathogenic and nonpathogenic mutations, respectively. *, $p < 0.05$.

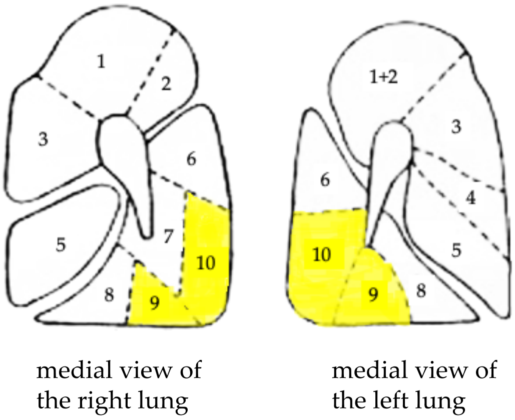

Figure S2. Explanatory schemes of the anatomical lung segments. Segments 9 and 10, the dorsal inferior lobes, were highlighted in yellow.

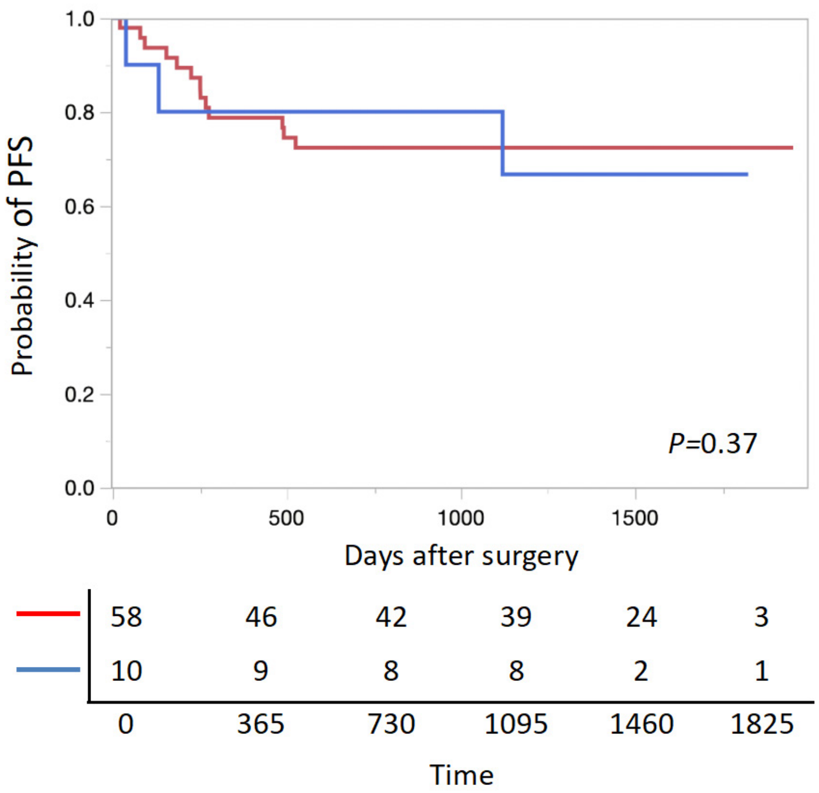

Figure S3. Locational variation in prognosis of lung cancers without *TP53* mutations. The prognosis was not significantly different between lung cancers without *TP53* mutations located in segments 9 and 10 and those located in segments 1 to 8. Blue and red lines denote tumors located in segments 9–10 and 1–8 (n = 10 and 58), respectively.

Table S1. Pathogenic variants identified in our assay.

	#_locus	type	ref	genotype	normal genotype	gene	transcript	function	codon	exon	protein	coding
1	chr17:7577539	SNV	G	G/A	G/G	TP53	NM_000546.5	missense	TGG	7	p.Arg248Trp	c.742C>T
2	chr10:89692985	SNV	G	G/T	G/G	PTEN	NM_000314.6	nonsense	TAA	5	p.Glu157Ter	c.469G>T
3	chr17:7578446	SNV	T	T/A	T/T	TP53	NM_000546.5	missense	TTC	5	p.Ile162Phe	c.484A>T
4	chr17:7578478	SNV	G	G/A	G/G	TP53	NM_000546.5	missense	CTC	5	p.Pro151Leu	c.452C>T
5	chr12:25398285	SNV	C	C/A	C/C	KRAS	NM_033360.3	missense	TGT	2	p.Gly12Cys	c.34G>T
6	chr12:56478947	SNV	C	C/T	C/C	ERBB3	NM_001982.3	missense	TGC	3	p.Arg135Cys	c.403C>T
7	chr19:11105657	SNV	G	G/A	G/G	SMARCA4	NM_001128849.1	missense	AAG	9	p.Glu525Lys	c.1573G>A
8	chr1:27106180	SNV	G	G/A	G/G	ARID1A	NM_006015.5	missense	AAG	20	p.Glu1931Lys	c.5791G>A
9	chr16:3820572	SNV	G	G/A	G/G	CREBBP	NM_004380.2	missense	CTG	14	p.Pro960Leu	c.2879C>T
10	chr13:49027160	SNV	C	C/T	C/C	RB1	NM_000321.2	missense	TTA	18	p.Ser576Leu	c.1727C>T
11	chr15:41961591	SNV	C	C/T	C/C	MGA	NM_001164273.1	missense	TAT	2	p.His167Tyr	c.499C>T
12	chr17:29528441	SNV	C	C/T	C/C	NF1	NM_001042492.2	nonsense	TAG	11	p.Gln400Ter	c.1198C>T
13	chr3:41266085	SNV	C	C/T	C/C	CTNNB1	NM_001904.3	nonsense	TAG	3	p.Gln28Ter	c.82C>T
14	chr12:46230632	SNV	G	G/A	G/G	ARID2	NM_152641.3	missense	AAA	8	p.Arg294Lys	c.881G>A
15	chr12:56481856	SNV	C	C/T	C/C	ERBB3	NM_001982.3	missense	TCT	7	p.Pro262Ser	c.784C>T
16	chr17:29687586	SNV	G	G/A	G/G	NF1	NM_001042492.2	missense	AAA	57	p.Glu2748Lys	c.8242G>A
17	chr3:41266118	SNV	G	G/A	G/G	CTNNB1	NM_001904.3	missense	ACC	3	p.Ala39Thr	c.115G>A
18	chr16:3831298	SNV	G	G/A	G/G	CREBBP	NM_004380.2	missense	CTA	7	p.Pro528Leu	c.1583C>T
19	chr7:116418932	SNV	G	G/A	G/G	MET	NM_001127500.2	missense	CAA	17	p.Arg1166Gln	c.3497G>A
20	chr12:46205250	SNV	G	G/A	G/G	ARID2	NM_152641.3	missense	AAG	4	p.Glu112Lys	c.334G>A
21	chr7:55268881	SNV	G	G/A	G/G	EGFR	NM_005228.4	missense	AGG	25	p.Gly983Arg	c.2947G>A
22	chr7:55241656	SNV	G	G/A	G/G	EGFR	NM_005228.4	missense	ACT	18	p.Ala702Thr	c.2104G>A
23	chr7:116381066	SNV	C	C/T	C/C	MET	NM_001127500.2	missense	CTT	5	p.Pro563Leu	c.1688C>T
24	chr1:243716098	SNV	G	G/A	G/G	AKT3	NM_005465.4	missense	TCT	10	p.Pro366Ser	c.1096C>T
25	chr16:3824662	SNV	T	T/C	T/T	CREBBP	NM_004380.2	missense	GAC	12	p.Asn731Asp	c.2191A>G
26	chr10:123247617	SNV	C	C/T	C/C	FGFR2	NM_000141.4	missense	CAA	14	p.Arg625Gln	c.1874G>A
27	chr17:29665110	SNV	C	C/T	C/C	NF1	NM_001042492.2	nonsense	TGA	45	p.Arg2258Ter	c.6772C>T
28	chr1:27092809	SNV	C	C/T	C/C	ARID1A	NM_006015.5	nonsense	TAG	9	p.Gln944Ter	c.2830C>T
29	chr17:29701087	SNV	C	C/T	C/C	NF1	NM_001042492.2	nonsense	TGA	58	p.Arg2812Ter	c.8434C>T
30	chr16:3778455	SNV	A	A/T	A/A	CREBBP	NM_004380.2	missense	CAG	31	p.Leu2198Gln	c.6593T>A
31	chr6:93967868	SNV	C	C/T	C/C	EPHA7	NM_004440.3	missense	AGG	11	p.Gly687Arg	c.2059G>A
32	chr9:139396309	SNV	C	C/T	C/C	NOTCH1	NM_017617.4	nonsense	TGA	30	p.Trp1843Ter	c.5529G>A
33	chr17:37881648	SNV	G	G/A	G/G	ERBB2	NM_004448.3	nonsense	TGA	22	p.Trp906Ter	c.2718G>A
34	chr6:93982080	SNV	G	G/A	G/G	EPHA7	NM_004440.3	missense	TTC	6	p.Ser462Phe	c.1385C>T
35	chr9:135781149	SNV	G	G/A	G/G	TSC1	NM_000368.4	missense	TAT	15	p.His606Tyr	c.1816C>T
36	chr17:29657406	SNV	C	C/T	C/C	NF1	NM_001042492.2	missense	CTT	39	p.Pro1901Leu	c.5702C>T
37	chr1:27087515	SNV	C	C/T	C/C	ARID1A	NM_006015.5	missense	TCG	5	p.Pro697Ser	c.2089C>T
38	chr11:118343316	SNV	G	G/A	G/G	KMT2A	NM_001197104.1	missense	AAC	3	p.Ser481Asn	c.1442G>A
39	chr12:25378571	SNV	C	C/T	C/C	KRAS	NM_033360.3	missense	AAA	4	p.Glu143Lys	c.427G>A
40	chr12:46243909	SNV	C	C/A	C/C	ARID2	NM_152641.3	missense	TAT	15	p.Ser668Tyr	c.2003C>A
41	chr16:2115635	SNV	A	A/G	A/A	TSC2	NM_000548.4	missense	CGG	16	p.Gln572Arg	c.1715A>G
42	chr7:55242464	INDEL	AGGAATTAAGAGAAGC	AGGAATTAAGAGAAGC/A	AGGAATTAAGAGAAGC/AGGAATTAAGAGAAGC	EGFR	NM_005228.3	nonframeshift Deletion	-	19	p.Glu746_Ala750del	c.2235_2249delGGAATTAAGAGAAGC
43	chr13:49050864	SNV	C	C/T	C/C	RB1	NM_000321.2	nonsense	TAG	25	p.Gln850Ter	c.2548C>T
44	chr13:48934194	SNV	C	C/T	C/C	RB1	NM_000321.2	nonsense	TAG	7	p.Gln217Ter	c.649C>T
45	chr19:1221247	SNV	G	G/A	G/G	STK11	NM_000455.4	missense	GAG	6	p.Gly257Glu	c.770G>A
46	chr17:7579355	SNV	A	A/G	A/A	TP53	NM_000546.5	missense	CCG	4	p.Leu111Pro	c.332T>C
47	chr17:7578242	SNV	C	C/A	C/C	TP53	NM_000546.5	missense	TTG	6	p.Val203Leu	c.607G>T
48	chr13:48881438	SNV	G	G/T	G/G	RB1	NM_000321.2	nonsense	TAA	2	p.Glu54Ter	c.160G>T
49	chr13:48955499	SNV	G	G/T	G/G	RB1	NM_000321.2	nonsense	TAA	17	p.Glu539Ter	c.1615G>T
50	chr17:7576897	SNV	G	G/A	G/G	TP53	NM_000546.5	nonsense	TAG	9	p.Gln317Ter	c.949C>T

51	chr19:10610247	SNV	C	C/A	C/C	KEAP1	NM_203500.1	missense	TTC	2	p.Val155Phe	c.463G>T
52	chr19:11098593	SNV	G	G/T	G/G	SMARCA4	NM_001128849.1	nonsense	TAG	6	p.Glu371Ter	c.1111G>T
53	chr18:48591919	SNV	G	G/A	G/G	SMAD4	NM_005359.5	missense	CAC	9	p.Arg361His	c.1082G>A
54	chr3:41280804	SNV	C	C/T	C/C	CTNNB1	NM_001904.3	nonsense	TAG	15	p.Gln773Ter	c.2317C>T
55	chr9:21971138	SNV	C	C/A	C/C	CDKN2A	NM_000077.4	missense	TAC	2	p.Asp74Tyr	c.220G>T
56	chr19:10610265	SNV	C	C/T	C/C	KEAP1	NM_203500.1	missense	AAG	2	p.Glu149Lys	c.445G>A
57	chr9:21971036	SNV	C	C/A	C/C	CDKN2A	NM_000077.4	missense	TAT	2	p.Asp108Tyr	c.322G>T
58	chr2:178098959	SNV	T	T/C	T/T	NFE2L2	NM_006164.4	missense	GGT	2	p.Asp29Gly	c.86A>G
59	chr13:48955538	SNV	C	C/T	C/C	RB1	NM_000321.2	nonsense	TGA	17	p.Arg552Ter	c.1654C>T
60	chr2:225360596	SNV	T	T/C	T/T	CUL3	NM_003590.4	missense	GTG	13	p.Met599Val	c.1795A>G
61	chr17:7577120	SNV	CG	CG/AG	CG/CG	TP53	NM_000546.5	missense	CTT	8	p.Arg273Leu	c.818G>T
62	chr17:7577553	SNV	ATGCAGGAACTGT	ATGCAGGAACTGT/ATGCAGGAACTGC	ATGCAGGAACTGT/ATGCAGGAACTGT	TP53	NM_000546.5	missense	AGC	7	p.Asn239Ser	c.716A>G
63	chr17:7578275	SNV	G	G/A	G/G	TP53	NM_000546.5	nonsense	TAG	6	p.Gln192Ter	c.574C>T
64	chr17:7578265	SNV	A	A/G	A/A	TP53	NM_000546.5	missense	ACC	6	p.Ile195Thr	c.584T>C
65	chr1:243736329	SNV	C	C/T	C/C	AKT3	NM_005465.4	missense	AAG	8	p.Glu240Lys	c.718G>A
66	chr17:7577550	SNV	C	C/A	C/C	TP53	NM_000546.5	missense	GTC	7	p.Gly244Val	c.731G>T
67	chr17:7578534	SNV	CTTG	CTTG/ATTG	CTTG/CTTG	TP53	NM_000546.5	missense	AAT	5	p.Lys132Asn	c.396G>T
68	chr17:7577546	SNV	GCC	GCC/GCT	GCC/GCC	TP53	NM_000546.5	missense	AGC	7	p.Gly245Ser	c.733G>A
69	chr11:108202607	SNV	T	T/C	T/T	ATM	NM_000051.3	missense	CCA	52	p.Leu2544Pro	c.7631T>C
70	chr22:41573941	SNV	G	G/T	G/G	EP300	NM_001429.3	missense	TCC	31	p.Ala2076Ser	c.6226G>T
71	chr2:178098960	SNV	C	C/T	C/C	NFE2L2	NM_006164.4	missense	AAT	2	p.Asp29Asn	c.85G>A
72	chr12:25398284	SNV	C	C/A	C/C	KRAS	NM_033360.3	missense	GTT	2	p.Gly12Val	c.35G>T
73	chr12:49426814	SNV	G	G/A	G/G	KMT2D	NM_003482.3	nonsense	TAG	39	p.Gln3892Ter	c.11674C>T
74	chr7:55241706	SNV	GG	GG/GT	GG/GG	EGFR	NM_005228.3	missense	TGC	18	p.Gly719Cys	c.2155G>T
75	chr17:7578280	SNV	G	G/A	G/G	TP53	NM_000546.5	missense	CTT	6	p.Pro190Leu	c.569C>T
76	chr17:7578526	SNV	CAA	CAA/CAT	CAA/CAA	TP53	NM_000546.5	missense	TTA	5	p.Phe134Leu	c.402T>A
77	chr17:7573976	SNV	T	T/A	T/T	TP53	NM_000546.5	nonsense	TAG	10	p.Lys351Ter	c.1051A>T
78	chr17:29677306	SNV	A	A/T	A/A	NF1	NM_001042492.2	missense	TTT	50	p.Tyr2476Phe	c.7427A>T
79	chr10:123353315	SNV	C	C/G	C/C	FGFR2	NM_000141.4	missense	CCT	2	p.Arg6Pro	c.17G>C
80	chr17:7577578	SNV	TGTA	TGTA/TTTA	TGTA/TGTA	TP53	NM_000546.5	nonsense	TAA	7	p.Tyr234Ter	c.702C>A
81	chr1:115256528	SNV	TTG	TTG/TTT	TTG/TTG	NRAS	NM_002524.4	missense	AAA	3	p.Gln61Lys	c.181C>A
82	chr3:41280704	SNV	G	G/A	G/G	CTNNB1	NM_001904.3	missense	ATA	15	p.Met739Ile	c.2217G>A
83	chr2:178098974	SNV	C	C/G	C/C	NFE2L2	NM_006164.4	missense	TCG	2	p.Trp24Ser	c.71G>C
84	chr12:56495023	SNV	G	G/A	G/G	ERBB3	NM_001982.3	missense	CAC	27	p.Arg1127His	c.3380G>A
85	chr17:7577544	SNV	AT	AT/AC	AT/AT	TP53	NM_000546.5	missense	GTG	7	p.Met246Val	c.736A>G
86	chr17:7578552	SNV	GT	GT/GC	GT/GT	TP53	NM_000546.5	missense	TGC	5	p.Tyr126Cys	c.377A>G
87	chr13:48941648	SNV	C	C/T	C/C	RB1	NM_000321.2	nonsense	TGA	10	p.Arg320Ter	c.958C>T
88	chr19:11130300	SNV	C	C/T	C/C	SMARCA4	NM_001128849.1	nonsense	TAG	18	p.Gln847Ter	c.2539C>T
89	chr17:7577022	SNV	G	G/A	G/G	TP53	NM_000546.5	nonsense	TGA	8	p.Arg306Ter	c.916C>T
90	chr17:7577514	SNV	G	G/T	G/G	TP53	NM_000546.5	missense	AAA	7	p.Thr256Lys	c.767C>A
91	chr17:37868208	SNV	C	C/T	C/C	ERBB2	NM_004448.3	missense	TTC	8	p.Ser310Phe	c.929C>T
92	chr17:7578475	SNV	G	G/A	G/G	TP53	NM_000546.5	missense	CTG	5	p.Pro152Leu	c.455C>T
93	chr17:7578406	SNV	C	C/A	C/C	TP53	NM_000546.5	missense	CTC	5	p.Arg175Leu	c.524G>T
94	chr3:178936091	SNV	G	G/A	G/G	PIK3CA	NM_006218.2	missense	AAG	10	p.Glu545Lys	c.1633G>A
95	chr19:10602878	SNV	G	G/A	G/G	KEAP1	NM_203500.1	missense	TGG	3	p.Arg234Trp	c.700C>T
96	chr16:3807902	SNV	G	G/A	G/G	CREBBP	NM_004380.2	nonsense	TGA	18	p.Arg1173Ter	c.3517C>T
97	chr17:7577108	SNV	C	C/A	C/C	TP53	NM_000546.5	missense	TTT	8	p.Cys277Phe	c.830G>T
98	chr2:178098816	SNV	C	C/A	C/C	NFE2L2	NM_006164.4	missense	TAT	2	p.Asp77Tyr	c.229G>T
99	chr17:7578452	SNV	TGGCGCGGAC	TGGCGCGGAC/TGGCGGGGAC	TGGCGCGGAC/TGGCGCGGAC	TP53	NM_000546.5	missense	CCC	5	p.Arg158Pro	c.473G>C
100	chr15:66727455	SNV	G	G/T	G/G	MAP2K1	NM_002755.3	missense	AAT	2	p.Lys57Asn	c.171G>T
101	chr12:25380275	SNV	T	T/G	T/T	KRAS	NM_033360.3	missense	CAC	3	p.Gln61His	c.183A>C
102	chr12:25398283	SNV	ACC	ACC/ACA	ACC/ACC	KRAS	NM_033360.3	missense	TGT|TGT	2	p.Gly12Cys	c.34G>T
103	chr2:178098804	SNV	C	C/T	./.	NFE2L2	NM_006164.4	missense	AGT	2	p.Gly81Ser	c.241G>A
104	chr19:10610154	SNV	C	C/T	C/C	KEAP1	NM_203500.1	missense	AGC	2	p.Gly186Ser	c.556G>A
105	chr19:1220644	SNV	C	C/T	C/C	STK11	NM_000455.4	missense	CTG	5	p.Pro221Leu	c.662C>T

106	chr17:7578190	SNV	T	T/C	T/T	TP53	NM_000546.5	missense	TGT	6	p.Tyr220Cys	c.659A>G
107	chr7:116412043	SNV	G	G/C	G/G	MET	NM_001127500.1	missense	CAT	14	p.Asp1028His	c.3082G>C
108	chr16:2134691	SNV	G	G/C	G/G	TSC2	NM_000548.3	missense	CAG	34	p.Glu1490Gln	c.4468G>C
109	chr15:42021449	SNV	G	G/A	G/G	MGA	NM_001164273.1	missense	AAG	11	p.Glu1249Lys	c.3745G>A
110	chr9:21971149	SNV	G	G/C	G/G	CDKN2A	NM_001195132.1	missense	CGC	2	p.Pro70Arg	c.209C>G
111	chr7:55242478	SNV	G	G/C	G/G	EGFR	NM_005228.3	missense	CCA	19	p.Ala750Pro	c.2248G>C
112	chr2:178098810	SNV	C	C/G	C/C	NFE2L2	NM_006164.4	missense	CAG	2	p.Glu79Gln	c.235G>C
113	chr17:7579406	SNV	G	G/C	./.	TP53	NM_000546.5	nonsense	TGA	4	p.Ser94Ter	c.281C>G
114	chr17:7578254	SNV	C	C/A	C/C	TP53	NM_000546.5	nonsense	TGA	6	p.Gly199Ter	c.595G>T
115	chr2:178098801	SNV	C	C/G	C/C	NFE2L2	NM_006164.4	missense	CAA	2	p.Glu82Gln	c.244G>C
116	chr7:55242466	INDEL	AGGAATTAAGAGAAGC	AGGAATTAAGAGAAGC/AGGAAGC	AGGAATTAAGAGAAGC/AGGAATTAAGAGAAGC	EGFR	NM_005228.3	nonframeshift Deletion	-	19	p.E746-R748del	c.2239_2247delTTAAGAGAA
117	chr7:140453179	SNV	C	C/T	C/C	BRAF	NM_004333.4	missense	AAA	15	p.Glu586Lys	c.1756G>A
118	chr7:55259515	SNV	T	T/G	T/T	EGFR	NM_005228.3	missense	CGG	21	p.Leu858Arg	c.2573T>G
119	chr2:178098944	SNV	C	C/A	C/C	NFE2L2	NM_006164.4	missense	CTA	2	p.Arg34Leu	c.101G>T
120	chr17:7578518	SNV	C	C/G	C/C	TP53	NM_000546.5	missense	CCC	5	p.Ala138Pro	c.412G>C
121	chr17:29664894	SNV	C	C/T	C/C	NF1	NM_001042492.2	nonsense	TAA	44	p.Gln2234Ter	c.6700C>T
122	chr7:140481402	SNV	CC	CC/GC	CC/CC	BRAF	NM_004333.4	missense	GCA	11	p.Gly469Ala	c.1406G>C
123	chr4:1805423	SNV	C	C/T	C/C	FGFR3	NM_000142.4	missense	GTG	8	p.Ala312Val	c.935C>T
124	chr2:225370756	SNV	C	C/G	C/C	CUL3	NM_003590.4	missense	CAG	8	p.Glu375Gln	c.1123G>C
125	chr17:7578469	SNV	CC	CC/AC	CC/CC	TP53	NM_000546.5	missense	GTC	5	p.Gly154Val	c.461G>T
126	chr17:29533316	SNV	G	G/A	G/G	NF1	NM_001042492.2	missense	CAA	12	p.Arg440Gln	c.1319G>A
127	chr19:1220629	SNV	C	C/T	C/C	STK11	NM_000455.4	missense	TTC	5	p.Ser216Phe	c.647C>T
128	chr12:56489541	SNV	G	G/A	G/G	ERBB3	NM_001982.3	missense	CAC	17	p.Arg669His	c.2006G>A
129	chr21:44524456	SNV	G	G/A	G/G	U2AF1	NM_006758.2	missense	TTT	2	p.Ser34Phe	c.101C>T
130	chr19:11141534	SNV	G	G/A	G/G	SMARCA4	NM_001128849.1	missense	ATG	25	p.Val1171Met	c.3511G>A
131	chr10:89720679	SNV	C	C/T	./.	PTEN	NM_000314.4	missense	ATA	8	p.Thr277Ile	c.830C>T
132	chr2:178098957	SNV	G	G/A	G/G	NFE2L2	NM_006164.4	missense	TTT	2	p.Leu30Phe	c.88C>T
133	chr1:120459031	SNV	C	C/T	C/C	NOTCH2	NM_024408.3	missense	CAG	34	p.Arg2105Gln	c.6314G>A
134	chr7:114304421	SNV	C	C/T	C/C	FOXP2	NM_148898.3	missense	TTC	17	p.Leu670Phe	c.2008C>T
135	chr19:1221976	SNV	G	G/T	G/G	STK11	NM_000455.4	missense	AGT	7	p.Arg297Ser	c.891G>T
136	chr19:11143994	SNV	G	G/A	G/G	SMARCA4	NM_001128849.1	missense	CAC	26	p.Arg1192His	c.3575G>A
137	chr3:41275779	SNV	G	G/T	G/G	CTNNB1	NM_001904.3	missense	CAT	10	p.Gln558His	c.1674G>T
138	chr7:55242465	INDEL	AGGAATTAAGAGAAGC	AGGAATTAAGAGAAGC/A	AGGAATTAAGAGAAGC/AGGAATTAAGAGAAGC	EGFR	NM_005228.3	nonframeshift Deletion	-	19	p.E746-A750del*	c.2235_2249delGGAATTAAGAGAAGC
139	chr22:41560092	SNV	A	A/T	A/A	EP300	NM_001429.3	missense	CTT	22	p.His1255Leu	c.3764A>T
140	chr18:48604750	SNV	G	G/T	G/G	SMAD4	NM_005359.5	missense	TGT	12	p.Trp524Cys	c.1572G>T
141	chr17:7574017	SNV	C	C/A	C/C	TP53	NM_000546.5	missense	CTC	10	p.Arg337Leu	c.1010G>T
142	chr3:178927980	SNV	T	T/C	./.	PIK3CA	NM_006218.2	missense	CGT	8	p.Cys420Arg	c.1258T>C
143	chr17:37880988	SNV	G	G/A	G/G	ERBB2	NM_004448.3	missense	ATG	20	p.Val773Met	c.2317G>A
144	chr3:178952085	SNV	A	A/T	A/A	PIK3CA	NM_006218.2	missense	CTT	21	p.His1047Leu	c.3140A>T
145	chr9:135771989	SNV	C	C/T	C/C	TSC1	NM_000368.4	missense	AAC	23	p.Ser1043Asn	c.3128G>A
146	chr17:7577528	SNV	GATGGGCCTCCGGTTC	GATGGGCCTCCGGTTC/GATGGGCCTCAGGTTC	GATGGGCCTCCGGTTC/GATGGGCCTCCGGTTC	TP53	NM_000546.5	missense	CTG	7	p.Arg248Leu	c.743G>T
147	chr19:10600447	SNV	G	G/A	G/G	KEAP1	NM_203500.1	missense	TGT	4	p.Arg470Cys	c.1408C>T
148	chr3:178936082	SNV	G	G/A	G/G	PIK3CA	NM_006218.3	missense	AAA	10	p.Glu542Lys	c.1624G>A
149	chr18:48584814	SNV	C	C/T	C/C	SMAD4	NM_005359.5	missense	TCC	7	p.Pro298Ser	c.892C>T
150	chr15:41991122	SNV	C	C/G	C/C	MGA	NM_001164273.1	nonsense	TGA	4	p.Ser692Ter	c.2075C>G
151	chr11:108143315	SNV	G	G/A	G/G	ATM	NM_000051.3	missense	TAC	21	p.Cys1045Tyr	c.3134G>A
152	chr9:139405140	SNV	C	C/T	C/C	NOTCH1	NM_017617.4	missense	CAC	17	p.Arg902His	c.2705G>A
153	chr2:225379486	SNV	G	G/A	G/G	CUL3	NM_003590.4	missense	TGT	4	p.Arg128Cys	c.382C>T
154	chr16:3781375	SNV	G	G/A	G/G	CREBBP	NM_004380.2	missense	TGC	30	p.Arg1664Cys	c.4990C>T
155	chr1:243828144	SNV	A	A/C	A/A	AKT3	NM_005465.4	missense	GTT	3	p.Phe72Val	c.214T>G
156	chr12:56495034	SNV	G	G/A	G/G	ERBB3	NM_001982.3	missense	ACC	27	p.Ala1131Thr	c.3391G>A
157	chr9:21971179	SNV	G	G/A	G/G	CDKN2A	NM_001195132.1	missense	GTG	2	p.Ala60Val	c.179C>T
158	chr1:27105920	SNV	G	G/A	G/G	ARID1A	NM_006015.4	nonsense	TAG	20	p.Trp1844Ter	c.5531G>A
159	chr10:89692792	SNV	C	C/A	C/C	PTEN	NM_000314.6	missense	GAA	5	p.Asp92Glu	c.276C>A
160	chr19:10602748	SNV	G	G/A	G/G	KEAP1	NM_203500.1	missense	ATG	3	p.Thr277Met	c.830C>T

161	chr17:7577094	SNV	GGTCTCT	GGTCTCT/CGTCTCT	GGTCTCT/GGTCTCT	TP53	NM_000546.5	missense	GGG	8	p.Arg282Gly	c.844C>G
162	chr9:139391715	SNV	C	C/T	C/C	NOTCH1	NM_017617.4	missense	CAC	34	p.Arg2159His	c.6476G>A
163	chr16:3788617	SNV	C	C/T	C/C	CREBBP	NM_004380.2	missense	CAC	26	p.Arg1446His	c.4337G>A
164	chr3:178922340	SNV	A	A/G	A/A	PIK3CA	NM_006218.2	missense	AGT	6	p.Asn370Ser	c.1109A>G
165	chr12:25380283	SNV	C	C/T	C/C	KRAS	NM_033360.3	missense	ACA	3	p.Ala59Thr	c.175G>A
166	chr12:46244889	SNV	C	C/T	C/C	ARID2	NM_152641.2	nonsense	TAA	15	p.Gln995Ter	c.2983C>T
167	chr22:41531892	SNV	C	C/T	C/C	EP300	NM_001429.3	missense	TTA	7	p.Ser535Leu	c.1604C>T
168	chr6:93967895	SNV	C	C/T	C/C	EPHA7	NM_004440.3	missense	AAC	11	p.Asp678Asn	c.2032G>A
169	chr12:49446160	SNV	C	C/T	C/C	KMT2D	NM_003482.3	missense	AAG	10	p.Glu436Lys	c.1306G>A
170	chr5:86672800	SNV	G	G/A	G/G	RASA1	NM_002890.2	missense	AAA	17	p.Glu763Lys	c.2287G>A
171	chr5:86686658	SNV	A	A/C	A/A	RASA1	NM_002890.2	missense	CAC	25	p.Gln1034His	c.3102A>C
172	chr12:46243449	SNV	G	G/A	G/G	ARID2	NM_152641.2	missense	CAG	14	p.Arg601Gln	c.1802G>A
173	chr11:108098524	SNV	C	C/T	C/C	ATM	NM_000051.3	missense	TGC	3	p.Arg32Cys	c.94C>T
174	chr3:178952074	SNV	G	G/A	G/G	PIK3CA	NM_006218.2	missense	ATA	21	p.Met1043Ile	c.3129G>A
175	chr9:21971017	SNV	G	G/A	G/G	CDKN2A	NM_000077.4	missense	CTC	2	p.Pro114Leu	c.341C>T
176	chr17:29553550	SNV	C	C/G	C/C	NF1	NM_001042492.2	missense	AGT	18	p.Thr700Ser	c.2099C>G
177	chr11:108205780	SNV	C	C/T	C/C	ATM	NM_000051.3	missense	TCA	55	p.Pro2699Ser	c.8095C>T
178	chr19:11141519	SNV	C	C/T	C/C	SMARCA4	NM_001128849.1	nonsense	TAG	25	p.Gln1166Ter	c.3496C>T
179	chr12:56490604	SNV	C	C/T	C/C	ERBB3	NM_001982.3	missense	TGG	19	p.Arg750Trp	c.2248C>T
180	chr9:139412673	SNV	G	G/A	./.	NOTCH1	NM_017617.3	missense	TCT	7	p.Pro391Ser	c.1171C>T
181	chr7:140453136	SNV	A	A/T	A/A	BRAF	NM_004333.4	missense	GAG	15	p.Val600Glu	c.1799T>A
182	chr7:140453133	SNV	TTCAC	TTCAC/TTCAA	TTCAC/TTCAC	BRAF	NM_004333.4	missense	TTG	15	p.Val600Leu	c.1798G>T
183	chr19:1220410	SNV	A	A/G	A/A	STK11	NM_000455.4	missense	CGT	4	p.His168Arg	c.503A>G
184	chr12:46246113	SNV	C	C/T	C/C	ARID2	NM_152641.2	nonsense	TAG	15	p.Gln1403Ter	c.4207C>T
185	chr12:46211461	SNV	C	C/T	C/C	ARID2	NM_152641.2	missense	TGT	5	p.Arg143Cys	c.427C>T
186	chr3:47158132	SNV	G	G/A	G/G	SETD2	NM_014159.6	missense	TGT	4	p.Arg1523Cys	c.4567C>T
187	chr3:41266113	SNV	C	C/T	C/C	CTNNB1	NM_001904.3	missense	TTT	3	p.Ser37Phe	c.110C>T
188	chr17:7578415	SNV	AC	AC/AA	AC/AC	TP53	NM_000546.5	missense	TTT	5	p.Val172Phe	c.514G>T
189	chr19:1221313	SNV	GCCCCCC	GCCCCCC/GCCCCCT	GCCCCCC/GCCCCCC	STK11	NM_000455.4	missense	CTG	6	p.Pro281Leu	c.842C>T
190	chr19:11141523	SNV	C	C/T	C/C	SMARCA4	NM_001128849.1	missense	TTG	25	p.Ser1167Leu	c.3500C>T
191	chr18:48591915	SNV	G	G/T	G/G	SMAD4	NM_005359.5	missense	TAT	9	p.Asp360Tyr	c.1078G>T
192	chr2:178098799	SNV	T	T/A	./.	NFE2L2	NM_006164.4	missense	GAT	2	p.Glu82Asp	c.246A>T
193	chr9:21974732	SNV	A	A/G	A/A	CDKN2A	NM_000077.4	missense	CCG	1	p.Leu32Pro	c.95T>C
194	chr17:37881006	SNV	T	T/C	T/T	ERBB2	NM_004448.3	missense	CCC	20	p.Ser779Pro	c.2335T>C
195	chr4:1807841	SNV	G	G/A	G/G	FGFR3	NM_000142.4	missense	ACA	14	p.Ala634Thr	c.1900G>A
196	chr3:47125794	SNV	G	G/A	G/G	SETD2	NM_014159.6	missense	TGC	12	p.Arg1826Cys	c.5476C>T
197	chr6:93956510	SNV	C	C/T	C/C	EPHA7	NM_004440.3	missense	AAG	15	p.Arg909Lys	c.2726G>A
198	chr19:40748476	SNV	T	T/C	./.	AKT2	NM_001626.5	missense	GTG	5	p.Met136Val	c.406A>G
199	chr10:89725099	SNV	G	G/A	G/G	PTEN	NM_000314.6	missense	AAT	9	p.Ser361Asn	c.1082G>A
200	chr4:20619271	SNV	G	G/A	G/G	SLIT2	NM_004787.3	missense	CAA	36	p.Arg1449Gln	c.4346G>A
201	chr17:7578256	SNV	TCCACTCGGATAAGATGCTGAGGAGGGG	TCCACTCGGATAAGATGCTGAGGAGGGG/TCCACTCGGATACGATGCTGAGGAGGGG	TCCACTCGGATAAGATGCTGAGGAGGGG/TCCACTCGGATAAGATGCTGAGGAGGGG	TP53	NM_000546.5	missense	CGT	6	p.Leu194Arg	c.581T>G
202	chr16:3779773	SNV	G	G/T	G/G	CREBBP	NM_004380.2	missense	ACA	31	p.Pro1759Thr	c.5275C>A
203	chr19:11098396	SNV	C	C/T	C/C	SMARCA4	NM_001128849.1	missense	CTG	6	p.Pro305Leu	c.914C>T
204	chr12:25398314	SNV	G	G/A	G/G	KRAS	NM_033360.3	missense	ATT	2	p.Thr2Ile	c.5C>T
205	chr2:225342966	SNV	C	C/T	C/C	CUL3	NM_003590.4	missense	CAG	15	p.Arg709Gln	c.2126G>A
206	chr1:27106703	SNV	C	C/T	C/C	ARID1A	NM_006015.4	missense	ATC	20	p.Thr2105Ile	c.6314C>T
207	chr7:51098538	SNV	G	G/C	G/G	COBL	NM_015198.3	missense	AGC	9	p.Thr492Ser	c.1475C>G
208	chr19:40741860	SNV	C	C/T	C/C	AKT2	NM_001626.5	missense	CAC	11	p.Arg371His	c.1112G>A
209	chr3:47161976	SNV	T	T/C	T/T	SETD2	NM_014159.6	missense	GAT	3	p.Asn1384Asp	c.4150A>G
210	chr3:178938934	SNV	G	G/A	G/G	PIK3CA	NM_006218.2	missense	AAA	14	p.Glu726Lys	c.2176G>A
211	chr19:10610423	SNV	T	T/C	T/T	KEAP1	NM_203500.1	missense	CGC	2	p.His96Arg	c.287A>G
212	chr9:139418229	SNV	C	C/T	C/C	NOTCH1	NM_017617.3	missense	AGC	3	p.Gly115Ser	c.343G>A
213	chr12:25378562	SNV	C	C/T	C/C	KRAS	NM_033360.3	missense	ACA	4	p.Ala146Thr	c.436G>A
214	chr7:55233052	SNV	G	G/A	G/G	EGFR	NM_005228.3	missense	GAA	15	p.Gly601Glu	c.1802G>A
215	chr15:42028520	SNV	G	G/A	G/G	MGA	NM_001164273.1	missense	AAC	13	p.Ser1353Asn	c.4058G>A

216	chr11:108186775	SNV	G	G/A	G/G	ATM	NM_000051.3	missense	ACC	42	p.Ala2045Thr	c.6133G>A
217	chr7:114299725	SNV	G	G/A	G/G	FOXP2	NM_148898.3	nonsense	TGA	14	p.Trp573Ter	c.1719G>A
218	chr17:29588851	SNV	C	C/G	C/C	NF1	NM_001042492.2	nonsense	TGA	35	p.Ser1567Ter	c.4700C>G
219	chr9:139399799	SNV	C	C/T	C/C	NOTCH1	NM_017617.4	missense	AAC	25	p.Asp1517Asn	c.4549G>A
220	chr9:139390656	SNV	G	G/A	G/G	NOTCH1	NM_017617.3	missense	CTG	34	p.Pro2512Leu	c.7535C>T
221	chr7:116415114	SNV	G	G/A	G/G	MET	NM_001127500.2	missense	ATG	15	p.Val1088Met	c.3262G>A
222	chr12:108169333	SNV	C	C/T	C/C	ASCL4	NM_203436.2	missense	ATG	1	p.Thr114Met	c.341C>T
223	chr15:42035146	SNV	C	C/T	C/C	MGA	NM_001164273.1	missense	ATC	15	p.Thr1663Ile	c.4988C>T
224	chr22:41536175	SNV	G	G/A	G/G	EP300	NM_001429.3	missense	ACT	9	p.Ala598Thr	c.1792G>A
225	chr15:41961399	SNV	C	C/T	C/C	MGA	NM_001164273.1	missense	TGC	2	p.Arg103Cys	c.307C>T
226	chr12:25398280	SNV	GCCACC	GCCACC/GCCACG	GCCACC/GCCACC	KRAS	NM_033360.3	missense	CGT	2	p.Gly12Arg	c.34G>C
227	chr1:27023954	SNV	C	C/T	C/C	ARID1A	NM_006015.4	nonsense	TAA	1	p.Gln354Ter	c.1060C>T
228	chr17:7578492	SNV	C	C/T	C/C	TP53	NM_000546.5	nonsense	TGA	5	p.Trp146Ter	c.438G>A
229	chr9:135772900	SNV	C	C/T	C/C	TSC1	NM_000368.4	missense	CAG	21	p.Arg908Gln	c.2723G>A
230	chr10:89692811	SNV	G	G/T	G/G	PTEN	NM_000314.4	nonsense	TAA	5	p.Glu99Ter	c.295G>T
231	chr17:7579377	SNV	G	G/A	./.	TP53	NM_000546.5	nonsense	TAG	4	p.Gln104Ter	c.310C>T
232	chr4:1808949	SNV	TGCCCCCGGCCCCACCCAGCAGTGGGGGCTCGCGGACGTGAAG	TGCCCCCGGCCCCACCCAGCAGTGGGGGCTCGCGGACGTGAAG/TGCCCCCGGCCCCACCCAGCAGTGGGGACTCGCGGACGTGAAG	TGCCCCCGGCCCCACCCAGCAGTGGGGGCTCGCGGACGTGAAG/TGCCCCCGGCCCCACCCAGCAGTGGGGGCTCGCGGACGTGAAG	FGFR3	NM_001163213.1	missense	GAC	18	p.Gly805Asp	c.2414G>A
233	chr7:140454006	SNV	G	G/T	G/G	BRAF	NM_004333.4	missense	CAA	14	p.His574Gln	c.1722C>A
234	chr15:42002945	SNV	G	G/C	G/G	MGA	NM_001164273.1	missense	CAT	8	p.Asp828His	c.2482G>C
235	chr10:89711935	SNV	C	C/T	C/C	PTEN	NM_000314.6	missense	TAT	6	p.His185Tyr	c.553C>T
236	chr5:86633834	SNV	G	G/A	./.	RASA1	NM_002890.2	missense	AAT	5	p.Asp315Asn	c.943G>A
237	chr6:94068121	SNV	G	G/A	G/G	EPHA7	NM_004440.3	missense	TGT	4	p.Arg281Cys	c.841C>T
238	chr1:120465007	SNV	T	T/A	T/T	NOTCH2	NM_024408.3	missense	TTT	28	p.Ile1689Phe	c.5065A>T
239	chr2:225362480	SNV	G	G/A	G/G	CUL3	NM_003590.4	missense	CTA	12	p.Pro566Leu	c.1697C>T
240	chr6:93982075	SNV	G	G/A	G/G	EPHA7	NM_004440.3	nonsense	TAG	6	p.Gln464Ter	c.1390C>T
241	chr12:49445568	SNV	G	G/A	G/G	KMT2D	NM_003482.3	missense	TTG	10	p.Ser633Leu	c.1898C>T
242	chr17:7577070	SNV	GGAGATTCTCTTCCTCTGTGC	GGAGATTCTCTTCCTCTGTGC/GGAGATTCTCTTCCTTTGTGC	GGAGATTCTCTTCCTCTGTGC/GGAGATTCTCTTCCTCTGTGC	TP53	NM_000546.5	missense	AAG	8	p.Glu285Lys	c.853G>A
243	chr7:51261249	SNV	G	G/A	./.	COBL	NM_015198.3	nonsense	TAG	3	p.Gln95Ter	c.283C>T
244	chr19:11097617	SNV	C	C/T	C/C	SMARCA4	NM_001128849.1	missense	TTG	5	p.Ser266Leu	c.797C>T
245	chr1:243668559	SNV	G	G/A	G/G	AKT3	NM_005465.4	nonsense	TGA	13	p.Arg478Ter	c.1432C>T
246	chr22:41533715	SNV	C	C/T	C/C	EP300	NM_001429.3	nonsense	TAA	8	p.Gln561Ter	c.1681C>T
247	chr17:29587407	SNV	C	C/T	C/C	NF1	NM_001042492.2	missense	TTT	34	p.Ser1484Phe	c.4451C>T
248	chr12:46246056	SNV	C	C/T	C/C	ARID2	NM_152641.2	missense	TAT	15	p.His1384Tyr	c.4150C>T
249	chr16:3828769	SNV	G	G/A	./.	CREBBP	NM_004380.2	missense	TGC	9	p.Arg625Cys	c.1873C>T
250	chr17:7577506	SNV	C	C/A	C/C	TP53	NM_000546.5	missense	TAC	7	p.Asp259Tyr	c.775G>T
251	chr1:120471614	SNV	G	G/A	G/G	NOTCH2	NM_024408.3	missense	TGT	23	p.Arg1293Cys	c.3877C>T
252	chr3:178947844	SNV	G	G/A	G/G	PIK3CA	NM_006218.3	missense	ACT	19	p.Ala907Thr	c.2719G>A
253	chr4:1808865	SNV	C	C/T	C/C	FGFR3	NM_000142.4	missense	CTT	18	p.Pro766Leu	c.2297C>T
254	chr10:89692901	SNV	G	G/A	G/G	PTEN	NM_000314.6	missense	AGA	5	p.Gly129Arg	c.385G>A
255	chr16:3779094	SNV	C	C/T	C/C	CREBBP	NM_004380.2	missense	CAC	31	p.Arg1985His	c.5954G>A
256	chr13:48951168	SNV	C	C/T	C/C	RB1	NM_000321.2	nonsense	TAG	13	p.Gln444Ter	c.1330C>T
257	chr21:44513298	SNV	C	C/T	C/C	U2AF1	NM_006758.2	missense	AGT	8	p.Gly213Ser	c.637G>A
258	chr12:49433620	SNV	G	G/A	G/G	KMT2D	NM_003482.3	nonsense	TGA	31	p.Arg2645Ter	c.7933C>T
259	chr16:2115545	SNV	C	C/T	C/C	TSC2	NM_000548.3	missense	CTC	16	p.Pro542Leu	c.1625C>T

260	chr3:41266064	SNV	GCTGTTAGTCACTGGCAGCAACAGTCTTACCTGGACT	GCTGTTAGTCACTGGCAGCAACAGTCTTACCTGGACT/GCTGTTAGTCACTGGCAGCAACAGTCTTACCTGGCT	GCTGTTAGTCACTGGCAGCAACAGTCTTACCTGGACT/GCTGTTAGTCACTGGCAGCAACAGTCTTACCTGGACT	CTNNB1	NM_001904.3	missense	GGC	3	p.Asp32Gly	c.95A>G
261	chr3:178917544	SNV	G	G/A	G/G	PIK3CA	NM_006218.3	missense	CAA	3	p.Arg140Gln	c.419G>A
262	chr22:41574655	SNV	C	C/A	./.	EP300	NM_001429.3	missense	ACT	31	p.Pro2314Thr	c.6940C>A
263	chr16:2136741	SNV	C	C/T	C/C	TSC2	NM_000548.4	missense	TAC	38	p.His1620Tyr	c.4858C>T
264	chr17:29509657	SNV	G	G/A	G/G	NF1	NM_001042492.2	missense	ATG	8	p.Val288Met	c.862G>A
265	chr7:55259427	SNV	G	G/A	G/G	EGFR	NM_005228.3	missense	AAG	21	p.Glu829Lys	c.2485G>A
266	chr12:46244334	SNV	G	G/C	G/G	ARID2	NM_152641.2	missense	CCA	15	p.Ala810Pro	c.2428G>C
267	chr12:49447881	SNV	G	G/A	G/G	KMT2D	NM_003482.3	missense	TGC	5	p.Arg185Cys	c.553C>T
268	chr16:3827632	SNV	G	G/A	G/G	CREBBP	NM_004380.2	missense	TGC	11	p.Arg714Cys	c.2140C>T
269	chr19:11129641	SNV	C	C/T	C/C	SMARCA4	NM_001128849.1	missense	TTC	17	p.Ser816Phe	c.2447C>T
270	chr17:29556200	SNV	G	G/A	./.	NF1	NM_001042492.2	missense	AAC	21	p.Ser856Asn	c.2567G>A
271	chr17:7578181	SNV	G	G/A	G/G	TP53	NM_000546.5	missense	CTT	6	p.Pro223Leu	c.668C>T
272	chr3:178928109	SNV	G	G/A	G/G	PIK3CA	NM_006218.2	missense	AGA	8	p.Gly463Arg	c.1387G>A
273	chr17:29664858	SNV	A	A/G	A/A	NF1	NM_001042492.2	missense	GCG	44	p.Thr2222Ala	c.6664A>G
274	chr3:41280636	SNV	C	C/T	C/C	CTNNB1	NM_001904.3	missense	TGT	15	p.Arg717Cys	c.2149C>T
275	chr14:105239875	SNV	G	G/A	G/G	AKT1	NM_001014431.1	missense	TGG	9	p.Arg249Trp	c.745C>T
276	chr4:20525665	SNV	G	G/A	G/G	SLIT2	NM_004787.3	missense	AAC	14	p.Asp435Asn	c.1303G>A
277	chr11:108151894	SNV	A	A/G	A/A	ATM	NM_000051.3	missense	AGG	24	p.Lys1192Arg	c.3575A>G
278	chr17:37872806	SNV	C	C/T	C/C	ERBB2	NM_004448.3	missense	CTG	14	p.Pro562Leu	c.1685C>T
279	chr18:48581285	SNV	A	A/G	A/A	SMAD4	NM_005359.5	missense	GCC	5	p.Thr197Ala	c.589A>G
280	chr11:108205768	SNV	G	G/A	G/G	ATM	NM_000051.3	missense	AGT	55	p.Gly2695Ser	c.8083G>A
281	chrX:47040962	SNV	C	C/T	C/C	RBM10	NM_001204468.1	missense	TGC	14	p.Arg563Cys	c.1687C>T
282	chr7:55241708	SNV	G	G/C	G/G	EGFR	NM_005228.3	missense	GCC	18	p.Gly719Ala	c.2156G>C
283	chr9:139410075	SNV	G	G/A	G/G	NOTCH1	NM_017617.3	missense	ATC	11	p.Thr588Ile	c.1763C>T
284	chr1:120458381	SNV	G	G/A	G/G	NOTCH2	NM_024408.3	missense	TCC	34	p.Pro2322Ser	c.6964C>T
285	chr12:49438030	SNV	G	G/A	G/G	KMT2D	NM_003482.3	missense	CTT	21	p.Pro1714Leu	c.5141C>T
286	chr22:41574664	SNV	C	C/T	C/C	EP300	NM_001429.3	missense	TGG	31	p.Arg2317Trp	c.6949C>T
287	chr17:7579416	SNV	AGGAGGGGGCTGGTGCAGGGGCCGCCGGTGTAGGAGCTGCTGGTGC	AGGAGGGGGCTGGTGCAGGGGCCGCCGGTGTAGGAGCTGCTGGTGC/AGGGGGCTAGTGCAGGGGCCGCCGGTGTAGGAGCTGCTGGTGC	AGGAGGGGGCTGGTGCAGGGGCCGCCGGTGTAGGAGCTGCTGGTGC/AGGGGGCTGGTGCAGGGGCCGCCGGTGTAGGAGCTGCTGGTGC	TP53	NM_000546.5	missense	CTA	4	p.Pro87Leu	c.260C>T
288	chr16:2129190	SNV	C	C/T	C/C	TSC2	NM_000548.4	missense	TCG	27	p.Pro1042Ser	c.3124C>T
289	chr22:41533685	SNV	C	C/T	C/C	EP300	NM_001429.3	missense	TCC	8	p.Pro551Ser	c.1651C>T
290	chr9:139399393	SNV	G	G/A	G/G	NOTCH1	NM_017617.4	nonsense	TAG	26	p.Gln1584Ter	c.4750C>T
291	chr19:1220496	SNV	G	G/A	G/G	STK11	NM_000455.4	missense	ATG	4	p.Val197Met	c.589G>A
292	chr11:108203561	SNV	G	G/A	G/G	ATM	NM_000051.3	missense	AAG	53	p.Glu2621Lys	c.7861G>A
293	chr16:3820669	SNV	G	G/A	G/G	CREBBP	NM_004380.2	missense	TCG	14	p.Pro928Ser	c.2782C>T
294	chr19:11121103	SNV	C	C/T	C/C	SMARCA4	NM_001128849.1	missense	TTT	15	p.Leu724Phe	c.2170C>T
295	chr12:49432015	SNV	G	G/A	G/G	KMT2D	NM_003482.3	missense	TTC	34	p.Leu3042Phe	c.9124C>T
296	chr4:1808916	SNV	CAG	CAG/CAA	CAG/CAG	FGFR3	NM_001163213.1	missense	AGG	18	p.Gly786Arg	c.2356G>A
297	chr11:108143259	SNV	G	G/A	G/G	ATM	NM_000051.3	nonsense	TGA	21	p.Trp1026Ter	c.3078G>A
298	chr3:41266927	SNV	C	C/T	C/C	CTNNB1	NM_001904.3	missense	TGT	5	p.Arg200Cys	c.598C>T
299	chr12:56478860	SNV	C	C/T	C/C	ERBB3	NM_001982.3	nonsense	TGA	3	p.Arg106Ter	c.316C>T
300	chr1:27089742	SNV	G	G/A	G/G	ARID1A	NM_006015.4	missense	ACC	8	p.Ala900Thr	c.2698G>A

301	chr11:108180945	SNV	G	G/A	G/G	ATM	NM_000051.3	missense	ATT	39	p.Val1941Ile	c.5821G>A
302	chr19:11141498	SNV	G	G/A	G/G	SMARCA4	NM_001128849.1	missense	AGG	25	p.Gly1159Arg	c.3475G>A
303	chr19:11113756	SNV	G	G/A	G/G	SMARCA4	NM_001128849.1	missense	ATG	12	p.Val622Met	c.1864G>A
304	chr17:7577551	SNV	C	C/T	C/C	TP53	NM_000546.5	missense	AGC	7	p.Gly244Ser	c.730G>A
305	chr22:41560094	SNV	C	C/T	C/C	EP300	NM_001429.3	nonsense	TAG	22	p.Gln1256Ter	c.3766C>T
306	chr16:3831230	SNV	G	G/T	G/G	CREBBP	NM_004380.2	missense	ATT	7	p.Leu551Ile	c.1651C>A
307	chr17:7577604	SNV	C	C/T	C/C	TP53	NM_000546.5	missense	GAC	7	p.Gly226Asp	c.677G>A
308	chr11:108199797	SNV	G	G/A	G/G	ATM	NM_000051.3	missense	AAA	49	p.Arg2380Lys	c.7139G>A
309	chr9:139395206	SNV	G	G/A	G/G	NOTCH1	NM_017617.3	missense	TTC	31	p.Ser1911Phe	c.5732C>T
310	chr1:27101090	SNV	C	C/T	C/C	ARID1A	NM_006015.4	nonsense	TAG	18	p.Gln1458Ter	c.4372C>T
311	chr12:46244770	SNV	C	C/T	C/C	ARID2	NM_152641.2	missense	CTA	15	p.Pro955Leu	c.2864C>T
312	chr11:118375261	SNV	G	G/A	G/G	KMT2A	NM_001197104.1	missense	GAT	27	p.Gly2885Asp	c.8654G>A
313	chr1:243668597	SNV	C	C/T	C/C	AKT3	NM_005465.4	missense	CAG	13	p.Arg465Gln	c.1394G>A
314	chr3:189604245	SNV	G	G/A	G/G	TP63	NM_003722.4	missense	AAC	11	p.Ser471Asn	c.1412G>A
315	chr1:120471812	SNV	C	C/T	C/C	NOTCH2	NM_024408.3	missense	AAT	23	p.Asp1227Asn	c.3679G>A
316	chr12:46244329	SNV	T	T/C	T/T	ARID2	NM_152641.2	missense	ACA	15	p.Ile808Thr	c.2423T>C
317	chr9:139395006	SNV	G	G/A	G/G	NOTCH1	NM_017617.3	nonsense	TAG	31	p.Gln1978Ter	c.5932C>T
318	chr3:178941873	SNV	A	A/G	A/A	PIK3CA	NM_006218.3	missense	CGG	15	p.Gln731Arg	c.2192A>G
319	chr1:120466273	SNV	G	G/A	G/G	NOTCH2	NM_024408.3	nonsense	TAG	26	p.Gln1616Ter	c.4846C>T
320	chr3:41275750	SNV	C	C/T	C/C	CTNNB1	NM_001904.3	missense	TGC	10	p.Arg549Cys	c.1645C>T
321	chr9:139399338	SNV	G	G/A	G/G	NOTCH1	NM_017617.3	missense	ATC	26	p.Thr1602Ile	c.4805C>T
322	chr3:189607197	SNV	C	C/T	C/C	TP63	NM_003722.4	missense	TCT	12	p.Pro526Ser	c.1576C>T
323	chr16:2108755	SNV	A	A/G	A/A	TSC2	NM_000548.3	missense	GTG	10	p.Met286Val	c.856A>G
324	chr12:49446075	SNV	G	G/A	G/G	KMT2D	NM_003482.3	missense	GTA	10	p.Ala464Val	c.1391C>T
325	chr22:41574040	SNV	G	G/A	G/G	EP300	NM_001429.3	missense	AGG	31	p.Gly2109Arg	c.6325G>A
326	chr7:140453137	SNV	C	C/T	C/C	BRAF	NM_004333.4	missense	ATG	15	p.Val600Met	c.1798G>A
327	chr12:56492572	SNV	G	G/T	G/G	ERBB3	NM_001982.3	missense	TGG	23	p.Gly908Trp	c.2722G>T
328	chr19:11129670	SNV	G	G/A	G/G	SMARCA4	NM_001128849.1	missense	ACC	17	p.Ala826Thr	c.2476G>A
329	chr15:41989061	SNV	G	G/A	G/G	MGA	NM_001164273.1	missense	CAA	3	p.Arg618Gln	c.1853G>A
330	chr3:41266906	SNV	C	C/T	C/C	CTNNB1	NM_001904.3	nonsense	TAG	5	p.Gln193Ter	c.577C>T
331	chr17:37866420	SNV	C	C/T	C/C	ERBB2	NM_004448.3	missense	GTC	6	p.Ala242Val	c.725C>T
332	chr12:49444366	SNV	G	G/A	G/G	KMT2D	NM_003482.3	missense	CTC	11	p.Pro1002Leu	c.3005C>T
333	chr16:2131719	SNV	G	G/A	G/G	TSC2	NM_000548.4	missense	CAG	31	p.Arg1245Gln	c.3734G>A
334	chr1:120461167	SNV	G	G/A	G/G	NOTCH2	NM_024408.3	missense	TGC	32	p.Arg1931Cys	c.5791C>T
335	chr11:108196144	SNV	G	G/A	G/G	ATM	NM_000051.3	missense	CAC	46	p.Arg2227His	c.6680G>A
336	chr19:10600364	SNV	C	C/T	C/C	KEAP1	NM_203500.1	nonsense	TGA	4	p.Trp497Ter	c.1491G>A
337	chr16:3779313	SNV	G	G/A	./.	CREBBP	NM_004380.2	missense	TTA	31	p.Ser1912Leu	c.5735C>T
338	chr17:29552264	SNV	C	C/T	C/C	NF1	NM_001042492.2	missense	TTT	17	p.Ser666Phe	c.1997C>T
339	chr13:48955452	SNV	T	T/A	T/T	RB1	NM_000321.2	nonsense	TAA	17	p.Leu523Ter	c.1568T>A
340	chr17:7578442	SNV	TA	TA/CA	TA/TA	TP53	NM_000546.5	missense	TGC	5	p.Tyr163Cys	c.488A>G
341	chr7:116435757	SNV	C	C/T	C/C	MET	NM_001127500.1	missense	TCT	20	p.Pro1301Ser	c.3901C>T
342	chr17:7579876	SNV	G	G/A	G/G	TP53	NM_000546.5	missense	TCT	2	p.Pro13Ser	c.37C>T
343	chr7:140481380	SNV	C	C/T	C/C	BRAF	NM_004333.4	nonsense	TGA	11	p.Trp476Ter	c.1428G>A
344	chr16:2136299	SNV	G	G/A	G/G	TSC2	NM_000548.3	missense	AAC	37	p.Asp1590Asn	c.4768G>A
345	chr12:49420847	SNV	G	G/A	G/G	KMT2D	NM_003482.3	missense	TCT	48	p.Pro4968Ser	c.14902C>T
346	chr3:47162636	SNV	G	G/A	G/G	SETD2	NM_014159.6	nonsense	TAG	3	p.Gln1164Ter	c.3490C>T
347	chr16:3778479	SNV	C	C/T	C/C	CREBBP	NM_004380.2	missense	CAA	31	p.Arg2190Gln	c.6569G>A
348	chr16:2121535	SNV	C	C/T	C/C	TSC2	NM_000548.3	missense	TGG	18	p.Arg622Trp	c.1864C>T
349	chr6:93982107	SNV	C	C/T	C/C	EPHA7	NM_004440.3	missense	AAA	6	p.Arg453Lys	c.1358G>A
350	chr16:2125886	SNV	C	C/T	C/C	TSC2	NM_000548.3	missense	TCC	23	p.Pro878Ser	c.2632C>T
351	chr7:116403126	SNV	C	C/T	C/C	MET	NM_001127500.2	missense	TTA	11	p.Ser814Leu	c.2441C>T
352	chr1:27101066	SNV	G	G/A	G/G	ARID1A	NM_006015.4	missense	AGC	18	p.Gly1450Ser	c.4348G>A
353	chr1:27106648	SNV	G	G/A	G/G	ARID1A	NM_006015.4	missense	AGA	20	p.Gly2087Arg	c.6259G>A
354	chr1:120462063	SNV	C	C/T	C/C	NOTCH2	NM_024408.3	missense	ACC	31	p.Ala1885Thr	c.5653G>A
355	chr9:139407900	SNV	C	C/T	C/C	NOTCH1	NM_017617.3	missense	GAC	14	p.Gly766Asp	c.2297G>A
356	chr16:3779787	SNV	C	C/T	C/C	CREBBP	NM_004380.2	missense	AAC	31	p.Ser1754Asn	c.5261G>A
357	chr1:120506301	SNV	C	C/T	C/C	NOTCH2	NM_024408.3	missense	TAC	11	p.Cys604Tyr	c.1811G>A
358	chr1:27101571	SNV	C	C/T	C/C	ARID1A	NM_006015.4	missense	CTC	18	p.Pro1618Leu	c.4853C>T
359	chr1:27106354	SNV	C	C/T	C/C	ARID1A	NM_006015.4	nonsense	TGA	20	p.Arg1989Ter	c.5965C>T
360	chr19:1220689	SNV	T	T/C	T/T	STK11	NM_000455.4	missense	GCG	5	p.Val236Ala	c.707T>C

361	chr3:41266130	SNV	G	G/A	G/G	CTNNB1	NM_001904.3	missense	ACT	3	p.Ala43Thr	c.127G>A
362	chr7:55266502	SNV	C	C/T	C/C	EGFR	NM_005228.3	missense	TGC	23	p.Arg932Cys	c.2794C>T
363	chr9:21971105	SNV	C	C/T	C/C	CDKN2A	NM_001195132.1	missense	ACT	2	p.Ala85Thr	c.253G>A
364	chr14:105246553	SNV	C	C/T	C/C	AKT1	NM_001014431.1	missense	GAG	3	p.Gly16Glu	c.47G>A
365	chr16:2126143	SNV	G	G/A	G/G	TSC2	NM_000548.4	missense	CAG	24	p.Arg905Gln	c.2714G>A
366	chr1:120462030	SNV	G	G/A	G/G	NOTCH2	NM_024408.3	missense	TTC	31	p.Leu1896Phe	c.5686C>T
367	chr17:7579882	SNV	C	C/G	C/C	TP53	NM_000546.5	missense	CAG	2	p.Glu11Gln	c.31G>C
368	chr17:37863308	SNV	C	C/T	C/C	ERBB2	NM_004448.3	missense	TGC	2	p.Arg47Cys	c.139C>T
369	chr3:41274899	SNV	G	G/A	G/G	CTNNB1	NM_001904.3	nonsense	TGA	8	p.Trp383Ter	c.1149G>A
370	chr12:49421101	SNV	C	C/T	C/C	KMT2D	NM_003482.3	missense	AAC	48	p.Ser4883Asn	c.14648G>A
371	chr9:21971098	SNV	C	C/T	C/C	CDKN2A	NM_001195132.1	missense	CAG	2	p.Arg87Gln	c.260G>A
372	chr1:27057947	SNV	C	C/T	C/C	ARID1A	NM_006015.4	missense	TTA	3	p.Ser552Leu	c.1655C>T
373	chr1:120539871	SNV	G	G/A	G/G	NOTCH2	NM_024408.3	missense	TTC	4	p.Ser167Phe	c.500C>T
374	chr1:27099397	SNV	C	C/T	C/C	ARID1A	NM_006015.4	nonsense	TAG	14	p.Gln1212Ter	c.3634C>T
375	chr19:11121131	SNV	C	C/T	C/C	SMARCA4	NM_001128849.1	missense	GTC	15	p.Ala733Val	c.2198C>T
376	chr4:20591290	SNV	G	G/A	G/G	SLIT2	NM_004787.3	missense	AAC	30	p.Asp1038Asn	c.3112G>A
377	chr8:38271477	SNV	C	C/T	C/C	FGFR1	NM_001174067.1	missense	ATG	18	p.Val782Met	c.2344G>A
378	chr7:140453142	SNV	G	G/A	G/G	BRAF	NM_004333.4	missense	GTT	15	p.Ala598Val	c.1793C>T
379	chr9:139402765	SNV	G	G/A	G/G	NOTCH1	NM_017617.4	missense	TGC	20	p.Arg1082Cys	c.3244C>T
380	chr1:120465043	SNV	G	G/A	G/G	NOTCH2	NM_024408.3	nonsense	TAG	28	p.Gln1677Ter	c.5029C>T
381	chr17:7578476	SNV	G	G/T	G/G	TP53	NM_000546.5	missense	ACG	5	p.Pro152Thr	c.454C>A
382	chr4:20490588	SNV	G	G/A	G/G	SLIT2	NM_004787.3	missense	CAA	8	p.Arg253Gln	c.758G>A
383	chr12:49421652	SNV	C	C/G	C/C	KMT2D	NM_003482.3	missense	CAC	47	p.Gln4859His	c.14577G>C
384	chr19:40741178	SNV	G	G/A	G/G	AKT2	NM_001626.5	nonsense	TAG	12	p.Gln419Ter	c.1255C>T
385	chr17:29553582	SNV	C	C/T	C/C	NF1	NM_001042492.2	missense	TGC	18	p.Arg711Cys	c.2131C>T
386	chr22:41573611	SNV	G	G/A	G/G	EP300	NM_001429.3	missense	AGT	31	p.Gly1966Ser	c.5896G>A
387	chr19:11105622	SNV	G	G/A	G/G	SMARCA4	NM_001128849.1	missense	CAG	9	p.Arg513Gln	c.1538G>A
388	chr11:118348807	SNV	C	C/T	C/C	KMT2A	NM_001197104.1	missense	TGG	5	p.Arg1154Trp	c.3460C>T
389	chr16:2134274	SNV	G	G/A	G/G	TSC2	NM_000548.4	missense	AAG	34	p.Glu1351Lys	c.4051G>A
390	chr13:48916766	SNV	G	G/A	G/G	RB1	NM_000321.2	nonsense	TAG	3	p.Trp99Ter	c.296G>A
391	chr7:55259554	SNV	C	C/G	C/C	EGFR	NM_005228.4	missense	GGA	21	p.Ala871Gly	c.2612C>G
392	chr15:41988745	SNV	G	G/A	G/G	MGA	NM_001164273.1	missense	AAA	3	p.Glu513Lys	c.1537G>A
393	chr1:27057884	SNV	C	C/T	C/C	ARID1A	NM_006015.4	missense	CTG	3	p.Pro531Leu	c.1592C>T
394	chr16:3808917	SNV	G	G/A	G/G	CREBBP	NM_004380.2	nonsense	TGA	17	p.Arg1103Ter	c.3307C>T
395	chr17:7577099	SNV	C	C/T	C/C	TP53	NM_000546.5	missense	AAA	8	p.Arg280Lys	c.839G>A
396	chr7:55242485	SNV	C	C/T	C/C	EGFR	NM_005228.3	missense	TTT	19	p.Ser752Phe	c.2255C>T
397	chr7:55241644	SNV	G	G/A	G/G	EGFR	NM_005228.3	missense	ACT	18	p.Ala698Thr	c.2092G>A
398	chr7:55249071	SNV	C	C/T	C/C	EGFR	NM_005228.3	missense	ATG	20	p.Thr790Met	c.2369C>T
399	chr11:108203577	INDEL	CTTATA	CTTATA/C	CTTATA/CTTATA	ATM	NM_000051.3	frameshiftDeletion	AGC	53	p.Ile2629fs	c.7878_7882delTTATA
400	chr17:7578526	INDEL	CAA	CAA/C	CAA/CAA	TP53	NM_000546.5	frameshiftDeletion	TTG	5	p.Phe134fs	c.402_403delTT
401	chr17:7578526	INDEL	CAA	CAA/C	CAA/CAA	TP53	NM_000546.5	frameshiftDeletion	TTG	5	p.Phe134fs	c.402_403delTT
402	chr1:27101116	INDEL	A	A/AC	A/A	ARID1A	NM_006015.4	frameshiftInsertion	TGG	18	p.Gly1469fs	c.4398_4399insC
403	chr10:89653795	INDEL	CATT	CATT/C	CATT/CATT	PTEN	NM_000314.4	nonframeshift Deletion	-	2	p.Ile32del	c.94_96delATT
404	chr15:66729095	INDEL	GGAGATC	GGAGATC/G	GGAGATC/GGAGATC	MAP2K1	NM_002755.3	nonframeshift Deletion	-	3	p.Glu102_Ile103del	c.304_309delGAGATC
405	chr17:7578463	INDEL	CG	CG/C	CG/CG	TP53	NM_000546.5	frameshiftDeletion	GCG	5	p.Arg156fs	c.466delC
406	chr17:7576918	INDEL	TG	TG/T	TG/TG	TP53	NM_000546.5	frameshiftDeletion	ACA	9	p.Asn310fs	c.927delC
407	chr17:7578397	INDEL	TG	TG/T	TG/TG	TP53	NM_000546.5	frameshiftDeletion	ACC	5	p.His178fs	c.532delC
408	chr17:7578475	INDEL	G	G/GT	G/G	TP53	NM_000546.5	frameshiftInsertion	CAC	5	p.Pro152fs	c.454_455insA
409	chr17:7577064	INDEL	TC	TC/T	TC/TC	TP53	NM_000546.5	frameshiftDeletion	AGC	8	p.Glu294fs	c.873delG
410	chr11:108203577	INDEL	CTTATA	CTTATA/C	./.	ATM	NM_000051.3	frameshiftDeletion	AGC	53	p.Ile2629fs	c.7878_7882delTTATA
411	chr17:7579315	INDEL	G	G/GC	G/G	TP53	NM_000546.5	frameshiftInsertion	TGG	4	p.Cys124fs	c.371_372insG
412	chr17:7579315	INDEL	G	G/GC	G/G	TP53	NM_000546.5	frameshiftInsertion	TGG	4	p.Cys124fs	c.371_372insG

Table S2. Occurrence of mutations according to the location of cancer.

	**Superior** (*n* = 84)	**Inferior** (*n* = 56)	*p* value
Presence/absence of *EGFR* mutation	19/65	15/41	*p* = 0.57
Presence/absence of *KRAS* mutation	15/69	14/42	*p* = 0.31
Presence/absence of *TP53* mutation	54/30	32/24	*p* = 0.40
	**Anterior** (*n* = 29)	**Posterior** (*n* = 59)	*p* value
Presence/absence of *EGFR* mutation	11/18	13/46	*p* = 0.12
Presence/absence *of KRAS* mutation	5/24	15/44	*p* = 0.38
Presence/absence of *TP53* mutation	15/14	33/26	*p* = 0.71

Table S3. The genes targeted in the cancer panel.

No	Gene Symbol	Chromosome	Number of Amplicons	Total Bases	Covered Bases	Overall Coverage
1	AKT1	chr14	26	1573	1497	95%
2	AKT2	chr19	27	1576	1543	98%
3	AKT3	chr1	30	1624	1624	100%
4	ARID1A	chr1	76	7058	6023	85%
5	ARID1B	chr6	75	6950	5965	86%
6	ARID2	chr12	71	5718	5643	99%
7	ASCL4	chr12	5	532	382	72%
8	ATM	chr11	147	9791	9439	96%
9	BRAF	chr7	37	2481	2224	90%
10	CDKN2A	chr9	9	962	612	64%
11	COBL	chr7	48	4151	3977	96%
12	CREBBP	chr16	96	7639	7071	93%
13	CTNNB1	chr3	32	2486	2486	100%
14	CUL3	chr2	42	2561	2495	97%
15	EGFR	chr7	60	4189	4135	99%
16	EP300	chr22	90	7555	7182	95%
17	EPHA7	chr6	44	3175	3154	99%
18	ERBB2	chr17	57	4080	3808	93%
19	ERBB3	chr12	59	4440	4374	99%
20	FGFR1	chr8	41	2825	2816	100%
21	FGFR2	chr10	43	2910	2842	98%
22	FGFR3	chr4	34	2752	2215	81%
23	FOXP2	chr7	36	2487	2469	99%
24	HRAS	chr11	11	683	683	100%
25	KEAP1	chr19	24	1925	1845	96%
26	KMT2D	chr12	192	17154	15854	92%
27	KRAS	chr12	10	737	681	92%
28	MAP2K1	chr15	18	1292	1239	96%
29	MET	chr7	59	4427	4396	99%
30	MGA	chr15	110	9428	9345	99%
31	MLL	chr11	144	12,279	11875	97%
32	NF1	chr17	136	9161	9023	99%
33	NFE2L2	chr2	23	1868	1826	98%
34	NOTCH1	chr9	99	8008	7078	88%
35	NOTCH2	chr1	101	7809	7539	97%
36	NRAS	chr1	9	610	610	100%
37	PIK3CA	chr3	50	3407	3282	96%
38	PTEN	chr10	18	1302	1223	94%
39	RASA1	chr5	55	3412	3216	94%
40	RB1	chr13	55	3057	2902	95%
41	RBM10	chrX	48	3228	3079	95%
42	RIT1	chr1	13	771	771	100%
43	SETD2	chr3	91	7905	7663	97%
44	SLIT2	chr4	76	4972	4854	98%
45	SMAD4	chr18	24	1769	1715	97%

46	SMARCA4	chr19	74	5399	5055	94%
47	SOX2	chr3	9	964	883	92%
48	STK11	chr19	23	1392	1343	97%
49	TP53	chr17	22	1383	1351	98%
50	TP63	chr3	34	2360	2227	94%
51	TSC1	chr9	49	3705	3603	97%
52	TSC2	chr16	92	5834	5677	97%
53	U2AF1	chr21	15	880	870	99%

© 2020 by the authors. Licensee MDPI, Basel, Switzerland. This article is an open access article distributed under the terms and conditions of the Creative Commons Attribution (CC BY) license (http://creativecommons.org/licenses/by/4.0/).
